# Multifocal Primary Pancreatic Adenocarcinoma With No Precursor Lesion: A Report of Two Cases

**DOI:** 10.7759/cureus.48642

**Published:** 2023-11-11

**Authors:** Abdullah Aloraini, Renad A Almutawa, Nasser A Almutawa, Elaf A Almusahel, Rema A Almutawa, Ahmed Alhumaidi, Ahmad Madkhali

**Affiliations:** 1 Department of Surgery, College of Medicine, King Saud University, Riyadh, SAU; 2 College of Medicine, King Saud University, Riyadh, SAU; 3 Anatomical Pathology, King Saud University, Riyadh, SAU; 4 Surgery, King Saud University Medical City, Riyadh, SAU

**Keywords:** total pancreatectomy, double pancreatic adenocarcinomas, diffuse pancreatic adenocarcinoma, multifocal pancreatic adenocarcinoma, pancreatic cancer

## Abstract

The aim of this clinical case report is to highlight the unusual presentation of pancreatic malignancy in which multiple foci of primary adenocarcinoma involving the body and tail of the pancreas are associated with another primary pancreatic mass of adenocarcinoma in the pancreatic head with no precursor lesions in two cases. A retrospective medical chart review was performed at a tertiary hospital in Riyadh, Saudi Arabia, to identify cases with confirmed multifocal pancreatic adenocarcinoma. Data collected include clinical evaluations and laboratory and imaging results. Informed consent was waived. There was no evidence of multifocal cancer on imaging. The unexpected intraoperative findings and pathology report necessitated a total pancreatectomy for both cases. A negative imaging does not rule out a multifocal pancreatic adenocarcinoma. Such awareness may help in the early detection of pancreatic cancer. Moreover, the presence of more than one primary cancer in one organ is a distinctive phenomena that needs further study.

## Introduction

Carcinoma of the pancreas is one of the most aggressive cancers with a fatal outcome most of the time. In fact, the five-year survival rate in the United States was 9.2% for those diagnosed between 2007 and 2051 [1]. In the Saudi population, the one-year survival rate was 39%, and the five-year survival rate was 10%; in comparison to the United States surveillance epidemiology, Saudi Arabia had a slightly better survival [2]. Age and stage at diagnosis were independent prognostic factors for survival, which emphasize the importance of early diagnosis, although there is no effective screening for pancreatic cancer yet [2].

Incidence and mortality of pancreatic cancer are increasing year by year all around the world. Rates of new cases are three to four times higher in developed countries, with the world’s highest rate being in Europe and North America (age standardized rates (ASRs) of 7.7 and 7.6 per 100,000, respectively), while the lowest rates were observed in Africa and South Central Asia (ASR of 2.2 and <2 per 100.000, respectively) [1]. Pancreatic cancer ranks as the 15th cancer in Saudi Arabia with an incidence rate being in the lower borderline of the worldwide statistics (overall ASR of 2.26 and 1.41 per 100,000 for males and females, respectively) [3]. However, it ranks 8th in cancer-related mortality in Saudi Arabia [4].

These significant differences in incidence rates among countries are attributed to the presence of risk factors, such as tobacco smoking, which is considered the most important risk factor. Some rare cases of double or multiple masses of primary pancreatic adenocarcinoma have been reported in the literature, but a mass of primary pancreatic adenocarcinoma that is not associated with precursor lesions presenting as primary pancreatic adenocarcinoma in the form of multiple foci is something that has been unknown till now.

## Case presentation

Case summary for patient 1

A 66-year-old man with a medical history of inferior myocardial infarction 20 years ago, diabetes, hypertension, and dyslipidemia presented to the clinic with painless jaundice for three weeks associated with foul-smelling stool, diarrhea, and weight loss. He lost 14 kg over four to six weeks. He has no history of fever, night sweats, loss of appetite, anorexia, pruritus, and urinary symptoms and no family history of pancreatic/biliary cancers. The patient quit smoking 20 years ago. He is also non-alcoholic.

On physical examination, the patient was jaundiced with a soft lax abdomen. No masses or tenderness was palpated with a negative Murphy's sign. The laboratory test results were normal except the deranged liver function test, which showed the total bilirubin (101.87 mg/dL), direct bilirubin (76.92 mg/dL), gamma-glutamyl transferase (GGT) (2,433 U/L), alanine transaminase (ALT) (275 U/L), aspartate transaminase (AST) (220 U/L), and alkaline phosphatase (ALP) (1,155 U/L). Tumor marker levels carcinoembryonic antigen (CEA) (39.6 µg/L, normal range is <3.8 µg/L) and cancer antigen (CA) 19-9 (3,976.15 U/mL, normal range is <27 U/ml) were elevated.

**Figure 1:**
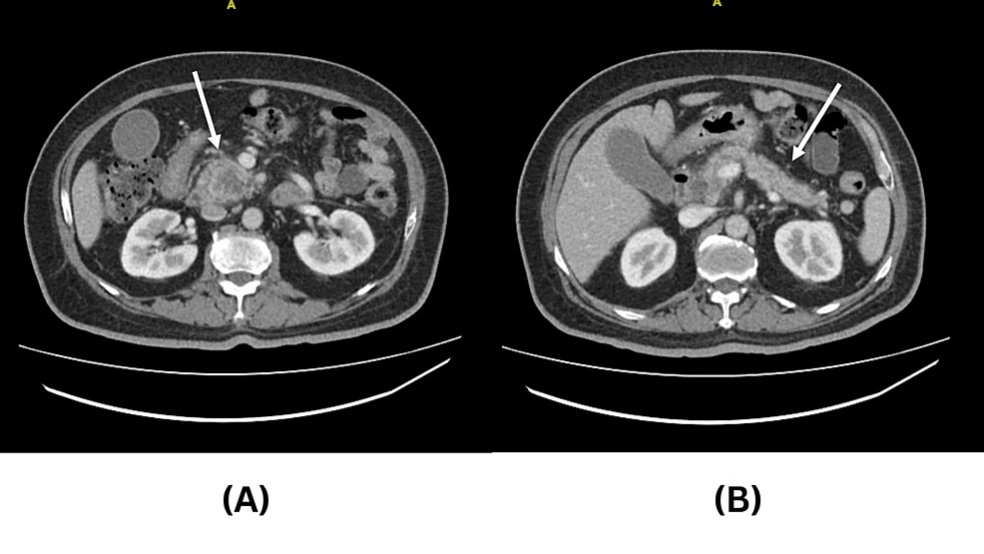
Axial CT scan for patient 1 (A) Axial CT showing the head/uncinate process of the pancreas with the tumor (arrow), (B) axial CT scan showing the body and tail of the pancreas clear of any lesion.

**Figure 2:**
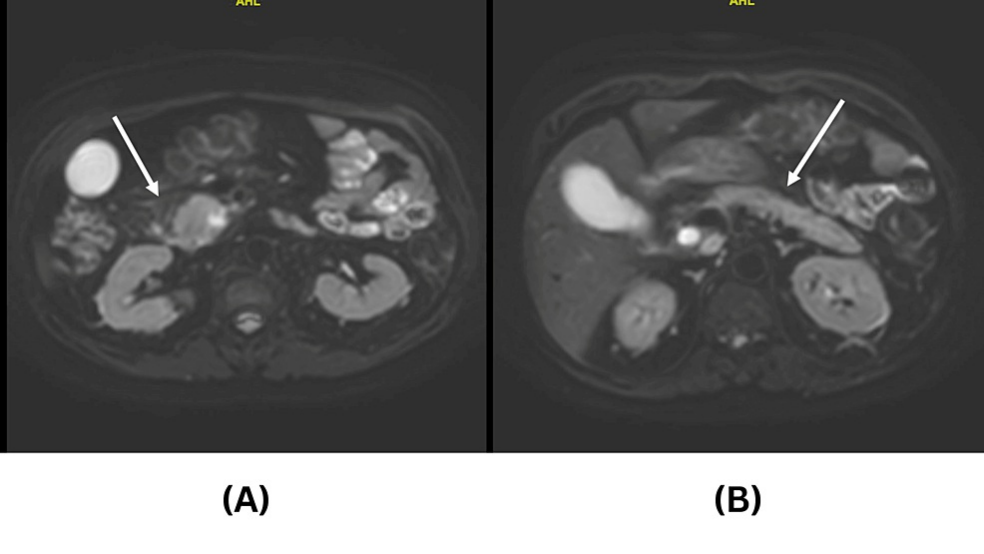
Axial MRI for patient 1 (A) Axial MRI-diffusion weighted imaging (DWI) showing the head of the pancreas mass (arrow), (B) axial MRI-DWI showing the body and tail of the pancreas both clear of any masses.

Abdominal CT revealed a 3 x 6 cm pancreatic head heterogeneous necrotic lesion with mild upstream pancreatic ductal dilation and moderate biliary dilation, as shown in Figure 1. Abdominal MRI revealed the presence of a localized lesion in the pancreas head and dilatation in the intra- and extrahepatic biliary trees (Figure 2). The findings are suggestive for pancreatic adenocarcinoma. No masses were seen on the body or tail of the pancreas. CT chest showed no intrathoracic metastasis. The patient was diagnosed to have a resectable pancreatic mass and was scheduled for a whipple procedure.

During the surgery, the pancreatic body margin was suspicious, so it was resected and sent for a frozen section, which came back positive for atypical cells with severe chronic pancreatitis, fibrosis, and inflammation. The second margin was positive for atypical cells, so the third frozen sections of the pancreatic margin were sent and reviewed, as malignant cells could not be ruled out. Since there were three frozen sections that were suspicious, with suspicious mass in the tail, the decision was made to go for total pancreatectomy and splenectomy. The patient received post-splenectomy vaccination on postoperative day 15 and was discharged on postoperative day 37.

**Figure 3:**
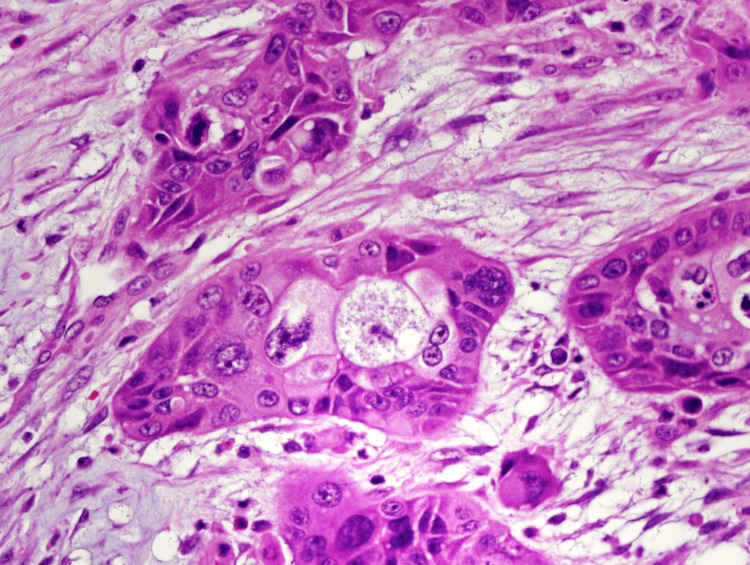
Histopathological section for patient 1 Photomicrograph of the histopathological section shows malignant glandular cells in the background of desmoplastic stoma (H-E stain; original magnification x400).

The histopathology examination of the permanent section of the specimen revealed invasive ductal adenocarcinoma moderately differentiated involving the head of the pancreas, with a size of 6.5 cm. Evidence of multiple foci in the body and tail pancreas was found. Perineural and lymphovascular invasions and peripancreatic tissue were positive for tumor extension. Five out of 45 lymph nodes were positive for metastatic adenocarcinoma. No precancerous lesions were found. The pTNM classification is pT3 N2 (Figure 3). Exclusion of involvement of the primary focus adenocarcinoma was achieved.

His postoperative period was complicated by heart failure, which he recovered well from. The patient received three cycles of gemcitabine and three cycles of cisplatin, but then died because of myocardial infarction eight months after the operation after completing his chemotherapy.

Case summary for patient 2

 

A 56-year-old female with known case of asthma and type 2 diabetes presented to the emergency department with jaundice, pruritus, abdominal pain for one week associated with one episode of vomiting, and fever that started one day ago. She denied any past medical, surgical, and family history. She is also non-alcoholic.

On physical examination, the patient was oriented to time, place, and person but looked sick and jaundiced; she was also found to be hypotensive (85/49 mmHg) and febrile with a temperature of 37.6 °C. On abdominal examination, there was tenderness all over the abdomen mainly in the right upper quadrant and right lower quadrant with a positive Murphy's sign. Laboratory tests revealed leukocytosis and deranged liver function test, which showed the total bilirubin (400 mg/dL), direct bilirubin (300 mg/dL), ALT (30 U/L), AST (20.7 U/L), and ALP(201.0 U/L). Tumor marker levels CEA (1.90 µg/L; normal range is <3.8 µg/L) and CA 19-9 (83.90 kU/L; normal range is <27 kU/L) were elevated.

**Figure 4:**
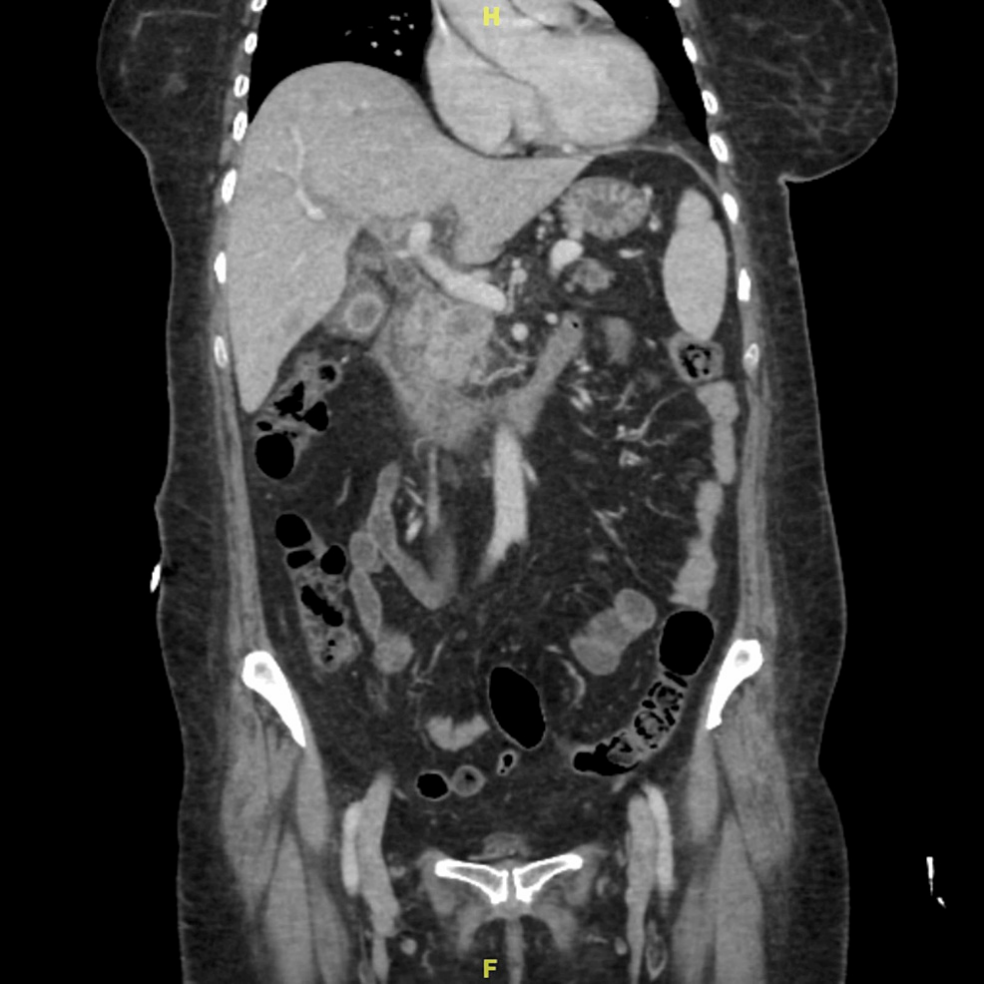
Coronal CT for patient 2 Coronal CT of abdomen and pelvis shows a pancreatic head mass.

**Figure 5:**
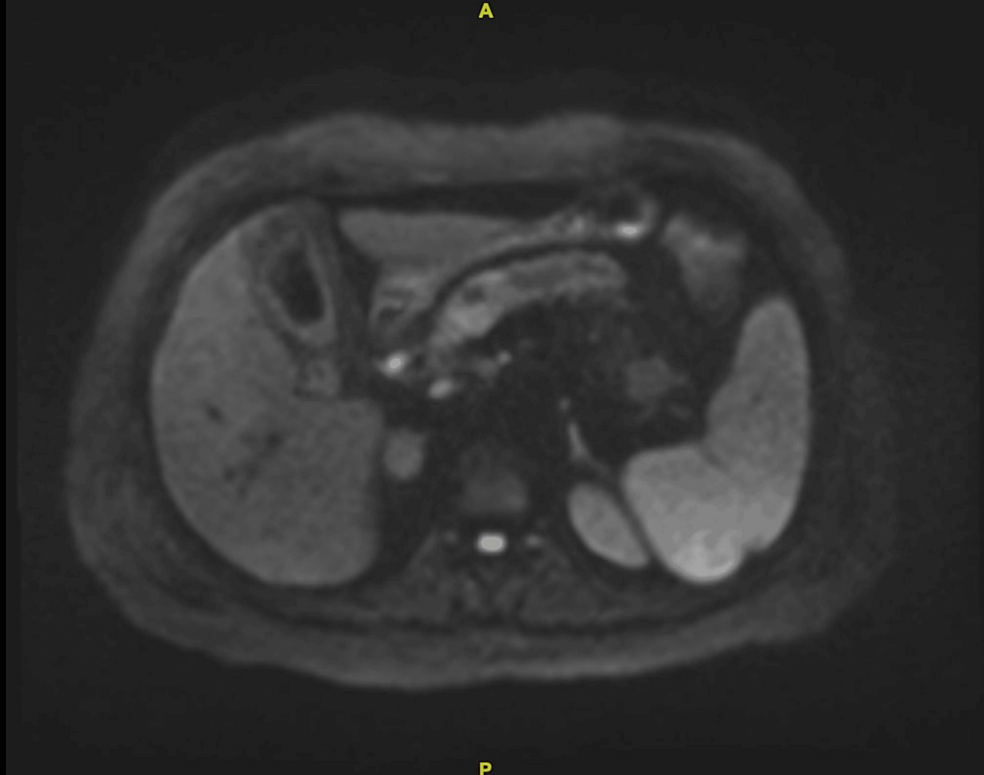
Axial MRI for patient 2 Axial MRI showing the body and tail of the pancreas with dilated ducts but no mass.

Abdominal CT scan was done and showed a pancreatic head mass and dilated intra- and extrahepatic biliary trees with multiple gallstones and no vascular invasion, as shown in Figure 4. The patient was admitted as a case of ascending cholangitis secondary to pancreatic head mass and underwent urgent endoscopic retrograde cholangiopancreatography (ERCP) with placement of a plastic stent. Further workup with MRI and magnetic resonance cholangiopancreatography (MRCP) showed the pancreatic head mass likely representing pancreatic adenocarcinoma with pancreatic duct dilatation and atrophy and mild intra- and extrahepatic biliary duct dilatation with an interval improvement of periductal inflammatory changes (Figure 5). No masses were seen on the body or tail of the pancreas. CT chest showed no intrathoracic metastasis. The patient was diagnosed to have a resectable pancreatic mass and was scheduled for a whipple procedure.

During the surgery, the head of the pancreas mass was invading the portal vein, and multiple hard masses were found in the tail and body of the pancreas. The findings were discussed with the family during the operation, and after their decision, the whipple procedure was changed to total pancreatectomy and splenectomy with portal vein resection and reconstruction. The patient received post-splenectomy vaccination and was discharged on postoperative day 12.

**Figure 6:**
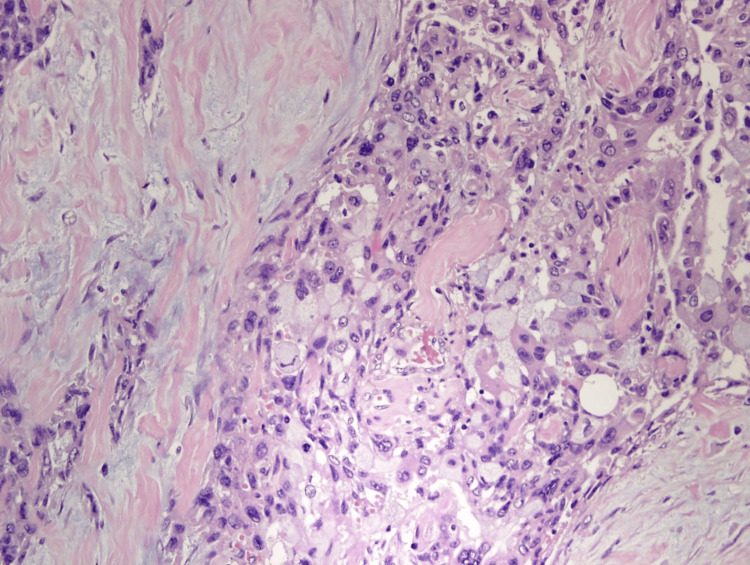
Histopathological section for patient 2 Photomicrograph of the histopathological section shows poorly differentiated malignant epithelial cells on the background of desmoplastic stroma (H-E stain; original magnification x400).

The histopathology examination of the specimen revealed invasive ductal adenocarcinoma, poorly differentiated, involving the head of the pancreas with a size of 5 cm with perineural and lymphovascular invasion. Five out of 53 lymph nodes were positive for metastatic adenocarcinoma. The uncinate margin was focally positive for tumor cells. There was a direct extension to the duodenal wall and peripancreatic soft tissue. No precancerous lesions and no evidence of chronic pancreatitis were found. The body and tail of the pancreas showed multifocal invasive ductal adenocarcinoma. The pTNM classification was pT3 N2 Mx (Figure 6). Exclusion of involvement by the primary focus adenocarcinoma was achieved.

The surgery was complicated by a wound infection that was managed as an outpatient. Unfortunately, the patient refused chemotherapy and died because of metastasis to the lung and liver six months after the operation.

## Discussion

Pancreatic adenocarcinoma in general has a wide variation of clinical presentations. Painless jaundice, progressive epigastric pain or signs of metastasis, or invasion of other structures are the most common presentations along with unintentional weight loss. Multifocal pancreatic adenocarcinoma diagnosis represents a challenge with the only lead being patient complaints.

The tumor marker level CA 19-9 was elevated in both cases, but both of them suffered from biliary obstruction and had hyperbilirubinemia, which affects the accuracy of the tumor marker interpretation, since it can also be elevated in benign biliary obstruction [5]. After obstruction relief, CA 19-9 can be used in the prognosis and follow-up of patients, but normal levels of CA 19-9 do not rule out malignancy [5]. The diagnostic pathology of pancreatic cancer is needed in borderline and unresectable masses in order to direct the management plan, and it is best obtained via endoscopic ultrasound-fine needle aspiration biopsy (EUS-FNAB). However, it is still not required for resectable masses, and imaging by CT and MRI is enough to proceed with surgery [6].

Possible findings of diffuse pancreatic adenocarcinoma in CT are peripheral capsule-like structure, invasion of vessels and neighbor organs, absence of the main pancreatic duct dilation within the tumor, and no parenchymal atrophy [7]. The multifocal form of adenocarcinoma could also mimic autoimmune pancreatitis on imaging as in a case reported by Miyoshi et al. [8] where they found a delayed enhanced sausage-shaped, enlarged pancreas, including a capsule-like rim at the surface and a dilated intrahepatic bile duct in enhanced CT; an EUS-FNAB was done that revealed a diagnosis of diffuse pancreatic adenocarcinoma. Thus, imaging is not always the right tool to rule out a multifocal or diffuse pancreatic adenocarcinoma, but a high index of suspicion is the key. In another case reported by Shohat et al. [9] of a 57-year-old female with abdominal pain and weight loss, the tumor markers were negative, abdominal CT revealed peripancreatic fluid collection, EUS-FNAB was negative, and surgery was performed. This case had no palpable pancreatic tumor, but two sizable peripancreatic fluid collections were discovered during surgery in the liver bed and the smaller sac, and the frozen section revealed multiple microscopic foci of adenocarcinoma with massive perineural and vascular invasion [9]. A persistent suspicion of cancer on the frozen section of the pancreatic margin and finding masses and nodules in the remnant pancreas with no previous history of pancreatitis were the triggers to proceed to total pancreatectomy in our patients. We believe that the presence of one or both of the above-mentioned intraoperative findings is needed to be assessed in every patient with pancreatic cancer to achieve oncologic resection in undetected multifocal disease on perioperative images.

Early diagnosis and treatment are essential for a better prognosis in pancreatic adenocarcinoma. Moreover, surgical resection is the most accounted curative treatment for these patients [9]. Total pancreatectomy is considered the best treatment for patients with multifocal pancreatic adenocarcinoma, especially if the head and tail of the pancreas are involved [10]. The strategy of treatment should be chosen carefully based on each patient with a consideration of undetectable masses preoperatively [10]. Fujimori et al. [10] proposed two theories regarding the development of the connected type of tumors. The first one is that these tumors have developed synchronously and independently without an extension and then extend to the pancreatic duct and communicate with one another [10]. The second theory is that the tumor is first developed in the pancreatic head, then extends along the pancreatic duct, and invades the distal pancreas distant from the main tumor [10].

In one study about the five-year survival rate after resections for pancreatic ductal adenocarcinoma, the five-year survival rate was 32.6% in node-negative patients, while it was 6.5% in node-positive patients with a 29.4% recurrence rate [11]. Longer overall survival was associated with the absence of lymph node infiltration, no blood transfusion, and early stage (I, IIa) [11]. Vascular invasion and postoperative complications were identified as significant independent predictors of survival [11]. Postoperatively, patients can experience metabolic consequences of a pancreatic state, including insulin-dependent diabetes, absence of functional glucagon, exocrine insufficiency, osteoporosis, and steatohepatitis with progressive liver failure secondary to decreased hepatic stimulation by glucagon [12].

**Figure 7:**
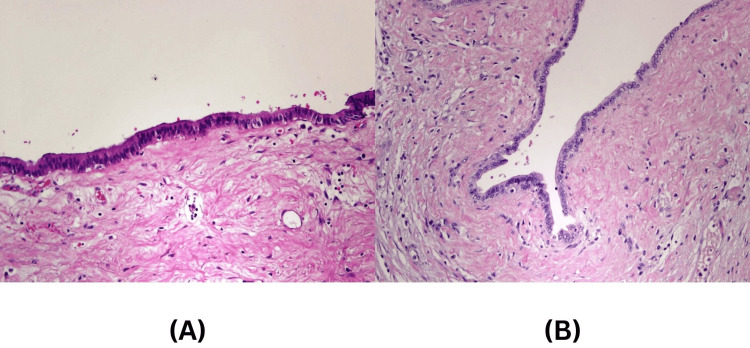
Pancreatic duct of the two patients Photomicrograph of the pancreatic duct reveals a single layer of benign columnar-cuboidal cell lining (H-E stain; original magnification x200 ). (a) The main pancreatic duct of the first patient, (b) the main pancreatic duct of the second patient.

Histological findings of multifocal adenocarcinoma usually reveals precancerous lesions (e.g., intraductal papillary mucinous neoplasms (IPMNs) [13-14] and pancreatic intraepithelial neoplasia (PanIN) [13]) giving chronic pancreatitis-like changes [13-14], but interestingly, in our two cases, there were no precancerous lesions identified. The pathogenesis of the multifocal pancreatic adenocarcinoma is still unknown. These lesions might be connected with one another or separated. Naito et al. [15] reported a case of multicentric development of the pancreatic body and tail carcinomas, but the histological findings of the main pancreatic duct showed that the main pancreatic duct between the two tumors was discontinuous. Moreover, multiple factors can support the first theory in our two cases, namely, absence of the dysplastic cells in the pancreatic duct (Figure 7) and absence of the precancerous lesions in both cases.

Table 1 summarizes the literature review. 

**Table 1:** Summary of the literature on multifoci pancreatic adenocarcinomas

Study	Age	Sex	Presentation	Radiographic study type	Radiographic findings	Histopathological examination	Tumor markers
Shohat et al. (2016) [9]	57 years	Female	Epigastric abdominal pain, weight loss	Abdominal CT, MRI, and endoscopic ultrasound (EUS)	Peripancreatic fluid collection	Evidence of massive perineural and vascular invasion, with several of them reaching the surgical margins at the pancreatic neck	Normal
Fujimori et al. (2022) [10]	61 years	Male	Elevated liver enzymes during a routine check-up for type 2 diabetes mellitus	Abdominal CT	Hypovascular mass in the pancreatic body with partial encasement of the common hepatic artery, left gastric artery, celiac artery, and splenic artery and invasion of the splenic vein	Pancreatic ductal adenocarcinoma (ypT3N1aM0 ypStage IIB/UICC 8th) with synchronous extrahepatic cholangiocarcinoma (ypT2N1M0 ypStage IIB/UICC 8th)	Normal
De Silva et al. (2017) [14]	65 years	Female	Progressive obstructive jaundice	Abdominal CT and MRI	Two tumors of the pancreas. The ampullary tumor (17×19 mm) with ill-defined margins invading the adjacent pancreatic tissue and the tumor in the tail of the pancreas (28×38 mm) with irregular margins.	Adenocarcinoma with tumor cells suspended in pools of extracellular mucin	Carcinoembryonic antigen (CEA) and epithelial membrane antigen (EMA) positivity is reported in both tumors.

## Conclusions

The importance of this report is to spread awareness of the possibility of the multifocal form of pancreatic adenocarcinoma that might not be shown on perioperative imaging. This raises the issue of the possibility of needing to investigate all pancreatic masses with EUS prior to surgery or routine intraoperative ultrasonography (IOUS). With such findings, it might be necessary to speak to all patients about converting such an operation to a more aggressive one.

In conclusion, a negative imaging does not rule out a multifocal pancreatic adenocarcinoma. Such awareness may help in the early detection of pancreatic cancer. Moreover, the presence of more than one primary cancer in one organ is a distinctive phenomena that needs further study.
